# A Cohort Study of Gastric Fluid and Urine Metabolomics for the Prediction of Survival in Severe Prematurity

**DOI:** 10.3390/metabo13060708

**Published:** 2023-05-30

**Authors:** Konstantia Besiri, Olga Begou, Olga Deda, Evmorfia Bataka, Christos Nakas, Helen Gika, Angeliki Kontou, Eleni Agakidou, Kosmas Sarafidis

**Affiliations:** 1 1st Department of Neonatology, School of Medicine, Aristotle University of Thessaloniki, 54124 Thessaloniki, Greece; kmpesiri@auth.gr (K.B.); angiekon2001@yahoo.gr (A.K.); eagakidou@auth.gr (E.A.); 2 School of Chemistry, Aristotle University of Thessaloniki, 54124 Thessaloniki, Greece; mpegolga@chem.auth.gr (O.B.); olgadeda@auth.gr (O.D.); 3 Biomic_AUTh, Center for Interdisciplinary Research and Innovation (CIRI-AUTH), Balkan Center, B1.4, 57001 Thermi, Greece; gkikae@auth.gr; 4 Laboratory of Biometry, University of Thessaly, N. Ionia, 38446 Volos, Greece; bataka@uth.gr (E.B.); cnakas@uth.gr (C.N.); 5 Department of Clinical Chemistry, Inselspital, Bern University Hospital, University of Bern, 3010 Bern, Switzerland; 6 Laboratory of Forensic Medicine and Toxicology, School of Medicine, Aristotle University of Thessaloniki, 54124 Thessaloniki, Greece

**Keywords:** gastric fluid, urine, metabolite, GC-MS, prematurity, survival

## Abstract

Predicting survival in very preterm infants is critical in clinical medicine and parent counseling. In this prospective cohort study involving 96 very preterm infants, we evaluated whether the metabolomic analysis of gastric fluid and urine samples obtained shortly after birth could predict survival in the first 3 and 15 days of life (DOL), as well as overall survival up to hospital discharge. Gas chromatography–mass spectrometry (GC-MS) profiling was used. Uni- and multivariate statistical analyses were conducted to evaluate significant metabolites and their prognostic value. Differences in several metabolites were identified between survivors and non-survivors at the time points of the study. Binary logistic regression showed that certain metabolites in gastric fluid, including arabitol, and succinic, erythronic and threonic acids, were associated with 15 DOL and overall survival. Gastric glyceric acid was also associated with 15 DOL survival. Urine glyceric acid could predict survival in the first 3 DOL and overall survival. In conclusion, non-surviving preterm infants exhibited a different metabolic profile compared with survivors, demonstrating significant discrimination with the use of GC-MS-based gastric fluid and urine analyses. The results of this study support the usefulness of metabolomics in developing survival biomarkers in very preterm infants.

## 1. Introduction

Prematurity is a significant public health issue and the leading cause of death in children under 5 years. In 2010, approximately 15 million neonates were born prematurely, comprising 11% of all livebirths worldwide, with preterm birth rates varying from about 5% in several European countries to 18% in Africa [1,2]. Very preterm infants (<32-week gestation) represent a small percentage of infants born prematurely (1.6% of all live births in the United States), but despite the significant progress in perinatal and neonatal care, the risk of severe morbidities and/or death in these infants is still high [3,4]. In this context, outcome prediction, especially regarding the risk of demise in very preterm infants, is important for both clinical medicine and parent counseling. Thus, several disease severity scores and survival models have been developed for this purpose [5,6,7].

Metabolomics is a bio-analytical approach aiming to achieve comprehensive profiling of small molecules (<1 kDa) that participate in chemical reactions within living organisms. It is used for the study of biochemistry and biomarker discovery in various medical conditions, including neonatal medicine, and it provides new insights into the underlying pathophysiological mechanisms, helping in the detection of new diagnostic–prognostic biomarkers [8]. In neonates, urine and blood metabolomics has been used to develop early biomarkers of several common mortality causes, including sepsis [9], necrotizing enterocolitis [10], bronchopulmonary dysplasia [11] and brain injury [12], and more recently, for the prediction of survival in extremely preterm infants at the limits of viability [13].

Amniotic fluid has been used to assess fetal lung maturation for many years, typically after determining the lecithin-to-sphingomyelin ratio and phosphatidylglycerol or lamellar body counts [14]. More recently, amniotic fluid was found to predict not only preterm delivery but the development of bronchopulmonary dysplasia, a severe complication of prematurity [15]. The fetus swallows large amounts of amniotic fluid daily. This is gradually emptied from the stomach, a process associated with gastric secretion normally observed following feeding after birth [16]. Nevertheless, studies have shown that inflammatory mediators found in amniotic fluid may also be present in the gastric fluid of premature neonates after birth [17,18]. Furthermore, prior to the 1970s, gastric aspirate culture was popular for documenting early-onset neonatal sepsis, and there has been a recent resurgence of scientific interest in this approach [19].

On this basis, we hypothesized that the gastric fluid of preterm neonates, early after birth, shows differences in the metabolic profile associated with the development of complications of prematurity and, ultimately, survival. We also considered that our hypothesis could be also extended to urine, with the two biological samples possibly complementing each other in terms of metabolite changes.

Therefore, the aim of this study was to evaluate the presence of metabolites in the gastric fluid and urine of preterm neonates born after ≤32 weeks of gestation, which could serve as predictors of survival at different time points of a period of hospitalization in the neonatal intensive care unit (NICU).

## 2. Materials and Methods

### 2.1. Study Population

This is a prospective, single-center study performed on inborn preterm neonates (≤32-week gestation) who were admitted to our level III NICU between 1 March 2017 and 31 December 2020. Neonates were ineligible for the present study if they were outborn; had known congenital infections, anomalies, or inborn error of metabolism; or suffered from severe perinatal depression necessitating chest compressions in the delivery room. Other exclusion criteria included refusal of parental consent, inadequate selection of gastric and urine samples, and physical absence of the researcher.

We recorded variables related to neonatal demographical characteristics (gestational age, birth weight sex and being small for gestational age), pregnancy (multiple gestation, chorioamnionitis, hypertension/pregnancy-induced hypertension, maternal prenatal steroids and MgSO_4_ administration), delivery mode, neonatal status at birth (Apgar score at 1 and 5 min), interventions in the delivery room (intubation) and in the NICU setting (invasive mechanical ventilation, exogenous surfactant and drugs for the treatment of arterial hypotension and patent ductus arteriosus), complications of prematurity (respiratory distress syndrome, confirmed early- and late-onset sepsis, air-leak syndromes, severe brain injury, necrotizing enterocolitis and bronchopulmonary dysplasia), and survival in the first 3 and 15 days of life (DOL) and up to hospital discharge in survivors. Moreover, we used the updated version of the score for neonatal acute physiology–perinatal extension (SNAPPE-II score) as predictor of mortality [5]. The definitions of the variables used in the present study are shown in Supplementary File S1.

#### 2.1.1. Sampling

Gastric fluid was collected from the enrolled neonates with a thin gastric tube during the first hour of life. Urine samples were obtained after the first urination and within the first 12 h of life using plastic bags or using a bladder catheter placed for clinical reasons. Urine samples were centrifuged, and supernatants were stored along with gastric fluid samples at −80 °C until the metabolomic analyses were conducted.

#### 2.1.2. Outcomes

The primary outcomes of interest in this study were survival in the first DOL 3 and DOL 15, and at NICU discharge (overall survival).

### 2.2. Analytical Techniques

#### 2.2.1. Chemicals and Reagents

Methanol (MeOH; LC-MS grade) was obtained from CHEM-LAB NV (Zedelgem, Belgium). Methoxyamine hydrochloride (ΜeOX), N-Methyl-N-(trimethylsilyl) trifluoroacetamide (MSTFA), trimethylchlorosilane (TMCS) and pyridine anhydrous were purchased from Sigma-Aldrich (Merck Darmstadt, Germany). Urease derived from Canavalia ensiformis (Jack bean) (15,000–50,000 units/g), Myristic acid-d_7_ and pentadecane were also obtained from Sigma-Aldrich (Merck Darmstadt, Germany).

#### 2.2.2. Sample Preparation

##### Gastric Fluid Samples

All samples were left to thaw at room temperature (21 °C) prior to analysis. First, 50 mL of gastric fluid sample was diluted with 10 μL of myristic acid-d_7_ (internal standard, IS) and 50 μL of ice-cold MeOH (−20 °C). The sample was vigorously vortex-mixed for 2 min and then centrifuged at 10,000 rpm for 15 min. Next, seventy microliters of clear supernatant was transferred into a clear Eppendorf tube and evaporated to dryness under a vacuum. Then, 25 μL of MeOX 2% pyridine was added, and the sample was vortex-mixed for 2 min and placed in a heating device for 90 min at 30 °C. Subsequently, 50 μL of MSTFA 1% TMCS was added, and the sample was heated for 30 min at 37 °C. A volume of 10 μL of pentadecane (injection standard, 100 mg/L) was added, and the sample was subjected to untargeted GC-MS analysis.

##### Urine Samples

Urine samples were left to thaw at room temperature; then, 10 μL of myristic acid-d_7_ (internal standard (IS)) and 10 μL of urease (0.01 g/mL) were added to 70 μL of sample. The sample was vortex-mixed for 2 min and incubated at 37 °C for 1 h. Two hundred microliters of MeOH was added, and the sample was shaken for 2 min and centrifuged at 10,000 rpm for 15 min. Then, the derivatization procedure was followed as described above for 70 μL of the supernatant.

A pooled quality control (QC) sample was also prepared by mixing equal volumes of all analyzed samples in each respective matrix. QC samples were analyzed at the beginning of each analytical batch and every ten real samples to evaluate the analytical precision.

#### 2.2.3. GC-MS Analysis

GC-MS analysis was performed using an Agilent 7890A Gas Chromatography system coupled with a 5975C MSD (Agilent Technologies, Santa Clara, CA, USA), which was also equipped with a PTV injector and a CTC autosampler. Separation was performed with a 30 m HP-5 ms UI (Agilent J&W) capillary column, with a film thickness of 0.25 μm and an i.d. of 0.25 μm. Back-flush elution was carried out in a 1.5 m deactivated column with a film thickness of 0.18 mm. The total run time was 30 min, followed by a 12 min back-flush run.

The initial oven temperature was set at 60 °C for 1 min and then increased to 300 °C at a 10 °C/min rate. It was then maintained at 300 °C for 6 min. The injection volume was set at 1 μL. The solvent delay was 6 min, while helium (99.999%) was used as the carrier gas at a flow rate of 3 mL/min. The injection system was performed in splitless mode, and the PTV injector temperature was increased from 270 °C to 350 °C.

MS was operated at electron impact ionization mode (EI; 70 eV), where the ion source temperature and transfer line temperature were set at 230 °C and 250 °C, respectively. All mass spectra were acquired in full scan mode between 50 and 600 amu. GAVIN was used to perform peak integration complementary to AMDIS for peak deconvolution and identification. Metabolite identification was based on Agilent Fiehn Library.

### 2.3. Statistical Analysis

Descriptive statistics were calculated using means and standard deviations for quantitative variables and proportions for categorical variables. Uni- and multivariate statistical analyses were conducted to evaluate metabolic profiles. Univariate analyses comparing infants with the positive or negative outcome of interest were performed using *t*-tests and binary logistic regression for standardized data of continuous variables with a binary outcome and chi-squared tests for categorical variables.

Initially, univariate analysis was conducted to explore the effect of gastric fluid and urine metabolites on the main outcomes. *p*-value adjustment was achieved using the Benjamini–Hochberg procedure [20].

Next, multiple logistic regression analysis, followed by ROC/AUC, was applied to study the effect of significant metabolites documented in the first step of the univariate analysis, adjusting for significant perinatal–neonatal characteristics. Multivariate analyses on metabolomics data included cluster analysis and PLS-DA using the mixOmics package [21].

The sample size for the study was estimated using the MetSizeR [22] package. Expecting a 10% proportion of significant metabolites and a 7:1 ratio for survivors relative to non-survivors for the second endpoint (D15), considered as an average, resulted in an optimal sample size of 11 subjects for the non-survivor group (compared with 71 subjects for the survivor group), with the FDR set at 5%.

Adjusted *p*-values < 0.05 were considered statistically significant. Statistical analysis was performed using R, version 4.1.2 (The R Foundation for Statistical Computing, Vienna, Austria).

## 3. Results

### 3.1. Study Population

One hundred and fourteen preterm neonates were initially included in the study. However, ultimately, only 96 of them were used due to inappropriate sample storage (n = 16) and a lack of adequate clinical data (n = 2). Table 1 shows differences in perinatal and neonatal characteristics of the infants who were alive (survivors) or not (non-survivors) at the three time points of the study. Of the studied subjects, 90 (93.7%) were alive on DOL 3, and 78 (81.2%), on DOL 15, while 75 infants (78.1%) survived to NICU discharge.

The results of the estimated AUCs for the assessment of the prognostic performance of perinatal/neonatal variables of interest in survival are presented in Table 2.

### 3.2. Metabolomic Analysis Results

Based on the metabolic profiles obtained from gastric fluid, univariate analysis results revealed several significant metabolites associated with the endpoints of interest (six for survival on DOL 15 and four for overall survival). The descriptive and AUC values are summarized in Table 3.

A similar approach was used for the assessment of urine metabolites. The descriptive and AUC values are summarized in Table 5.

The results of the PLS-DA and hierarchical clustering of the full metabolic profiles, represented as subject projections onto a 2D space and as heatmaps, show a visual separation between survivors and non-survivors at the endpoints of interest, which is consistent with the amount of separation estimated based on univariate and multivariate data analyses. Separation was moderate, as illustrated in Figures S1–S10.

## 4. Discussion

In this prospective study, we evaluated whether metabolomic analyses (GC-MS) of gastric fluid and urine samples obtained soon after birth could potentially predict survival at different time points of postnatal life and up to NICU discharge in a cohort of very preterm infants.

Logistic regression revealed that certain metabolites in gastric fluid, including arabitol, and succinic, erythronic, and threonic acids, could predict 15 DOL and overall survival. Gastric glyceric acid could also predict 15 DOL survival. Moreover, glyceric acid in the urine could predict 3 DOL and overall survival.

### 4.1. Clinical Prediction of Survival in Preterm Neonates

Ensuring the survival of premature neonates receiving intensive care is a major challenge in everyday clinical practice. In a selected review of the mortality rates of neonatal intensive care units, the overall mortality rate of very preterm neonates ranged from 8% in China (October 2010 and September 2011) to 18.8% in Italy (2005) [23]. To this end, various scoring systems have been developed, as previously mentioned, to predict ΝICU mortality in very and extremely preterm infants, either based on specific gestational age thresholds, or to achieve a more accurate estimation, considering additional demographic, perinatal and clinical factors. The CRIB and SNAPPE-II scores or the outcome calculator developed by Tyson et al. [5,6,7] are examples of the illness severity and mortality risk scores commonly used by neonatologists. Interestingly, very premature infants have a longer lifespan than all NICU patients, a fact attributed to the substantial improvement in perinatal–neonatal care that allows infants to survive the immediate complications of prematurity but does not ensure survival from further complications [23]. This fact highlights the importance of predicting neonatal survival in different time periods during hospitalization, and not just that immediately after birth.

### 4.2. Use of Metabolomics to Prognosticate Survival of Preterm Neonates

In this investigation, we were able to discriminate surviving and non-surviving infants using untargeted metabolomics analyses (GC-MS) of two biological specimens: gastric fluid and urine, in which significant alternations were documented in various metabolites. Moreover, multiple logistic regression analysis allowed the identification of metabolites with significant prognostic value in terms of survival, even after adjusting for perinatal–neonatal characteristics. Pregnancy-related pathologies [24], the degree of prematurity [25] and medical problems in preterm infants [9] may significantly affect metabolic pathways in the mother and her child before and after birth.

There are very few data correlating metabolic profiles with neonatal survival. In a nested case–control study involving 465 singleton live births at 22–25 weeks of gestation, a strong association between metabolic patterns and infant 7-day survival (AUC 0.885, 95% CI 0.851–0.920) was documented. In the latter study, mass spectrometry (tandem mass spectrometry) was used to measure the amino acids, acylcarnitines and free carnitine in samples obtained as part of newborn screening. Low concentrations of alanine and high concentrations of ornithine were reported to be the two biggest correlates of survival in the model applied [13].

In the present study, five gastric fluid metabolites (arabitol, and succinic, erythronic, threonic and glyceric acids) and glyceric acid in the urine—all of which were significantly increased in non-survivors compared with surviving infants—were identified as significant predictors of survival. According to ROC analysis and the AUC values obtained, gastric fluid arabitol, and succinic acid, erythronic and threonic acid were found to better indicate survival on day of life 15 (AUC values of 0.863, 0.824, 0.841 and 0.845, for the respective metabolites). Urine glyceric acid was found to have AUC values ranging between 0.805 and 0.863. Interestingly, of the studied clinical parameters, gestational age, birth weight and the SNAPPE-II score were also found to be good-to-very good predictors of survival in our study population.

### 4.3. Interpretation of the Metabolic Alternations

Arabitol is a monosaccharide formed with the reduction of the pentose analogues [26]. Very little is known about polyol synthesis and its role in the fetus and neonates. High polyol concentrations have been observed during fetal life, possibly playing a protective role against the low-oxygen environment [27] and regulating the fluid balance by acting as organic osmolytes [28]. High urinary excretion of pentose phosphate pathway-associated polyols was reported after birth in both preterm and term neonates [29], although other studies showed significantly decreased polyols (mannitol/sorbitol arabitol/xylitol and xylose) in the urine of neonates with necrotizing enterocolitis [10] and sepsis [30]. Interestingly, increased polyols including arabitol in the urine and other body fluids have been described in inborn errors of the pentose phosphate pathway involving deficiencies in ribose 5-phosphate isomerase or transaldolase [26,31].

Patients with transaldolase deficiency also have high urinary concentrations of erythronic acid [31]. Notably, the latter polyol was significantly higher in the urine of non-survivors than in that of survivors. The higher (although not significantly different) incidence of severe intraventricular hemorrhage in infants who died compared with those who survived (33.3% vs. 17.8%) could provide an explanation for this finding. Recent data indicate circulating erythronic acid as an important indicator of traumatic brain injury in humans [32].

Threonic acid, a sugar with the same structure as erythronic acid *(*diastereomer),** was significantly higher in the gastric fluid of non-surviving infants than in that of surviving infants, supporting the use of this metabolite as a possible biomarker for neonatal mortality. Threonic acid is derived from ascorbic acid [33]. However, recent data show that threonic acid may also be produced by certain bacterial species of the intestinal microbiota (*Mucispirillum),* influencing its levels in urine [34]. Moreover, in an experimental model, an approximately threefold increase in serum threonic acid was observed four hours after the commencement of intestinal ischemia in the mouse. This fact was attributed to the oxidation of endogenous/dietary ascorbic acid under conditions of oxidative stress via an inflammatory cascade in response to ischemia [35].

Glyceric acid is an organic acid that we found to predict survival in very premature infants when detected in gastric fluid and in urine. Very little is known regarding its role in the neonatal period. In a recent study by Li et al. evaluating differences in fecal metabolites in normal infants and in those with breast milk jaundice, glyceric acid was reported among the major differentiating metabolites between the study groups [36]. Additionally, glyceric acid is excessively secreted in the urine of patients suffering from inborn errors of metabolism such as D- and L-glyceric aciduria [37].

Finally, succinate is a key intermediate in the tricarboxylic acid cycle (Krebs’ cycle), a primary metabolic pathway used to produce energy in the presence of oxygen. Altered succinate concentrations have been described in neonatal asphyxia/hypoxic–ischemic encephalopathy [38]. Interestingly, the selective accumulation of succinate in tissues is thought to be responsible for mitochondrial reactive oxygen species production and injury during reperfusion [39]. Oxidative stress is a major contributor to many important neonatal morbidities, especially in preterm infants [40], and consequently to increased mortality in this age group.

### 4.4. Advantages and Limitations of the Study

The present study has important advantages. To the best of our knowledge, this is the first study in which gastric fluid was used for metabolomic analysis in neonates. Μass spectrometry has been used in the development of rapid tests for the prediction of respiratory distress syndrome in neonates using gastric fluid aspirates, but only lecithin and sphingomyelin in the lung are measured [41]. Moreover, in contrast with a previously published relevant study evaluating 7-day survival [13], metabolic alternations were associated with survival at different time points and with hospital discharge, while several perinatal–neonatal characteristics associated with the subsequent clinical course during NICU stay were considered.

Nevertheless, this study has its limitations. For example, this was a pilot study conducted in a single center, limiting the number of neonates that could have been enrolled. Additionally, as previously stated, some of the discriminant metabolites observed in the present study (e.g., threonic acid) have been related to intestinal bacterial metabolism. The stomach–intestine of the newborns might have been colonized with microorganisms, prenatally, following an intra-amniotic infection, which is highly possible given its recognized correlation with or potential causative role in preterm birth [42]. In our investigation, around half of the infants studied were born to mothers with clinical or histological chorioamnionitis, and two had early-onset sepsis (classically attributed to microbes of maternal origin). Therefore, the inclusion of information on gut microbiota would have strengthened our findings by exploring the possible microbial origin of metabolites. In any case, the results of the present study should be validated in the context of large multicenter studies in the future. Only then will we be able to determine whether neonatal survival can be reliably predicted using metabolomic analysis of gastric fluid or urine obtained soon after birth. The generalizability of our findings should also be evaluated, considering other important factors such as the NICU setting as well as the interventions and/or treatments applied to the infants, in addition to maternal–infant characteristics. Overall, the role of metabolomics in predicting specific neonatal morbidities is a topic of great scientific interest that merits further investigation.

## 5. Conclusions

In conclusion, non-surviving preterm infants showed a different metabolic profile compared with survivors, allowing discrimination to be performed with the use of GC-MS-based gastric fluid and urine analyses. The results of this pilot study support the usefulness of metabolomics in developing novel early prognostic biomarkers of survival in very preterm infants. Gastric fluid seems to be a promising biological specimen for metabolomic research in neonates.

## Figures and Tables

**Figure 1:**
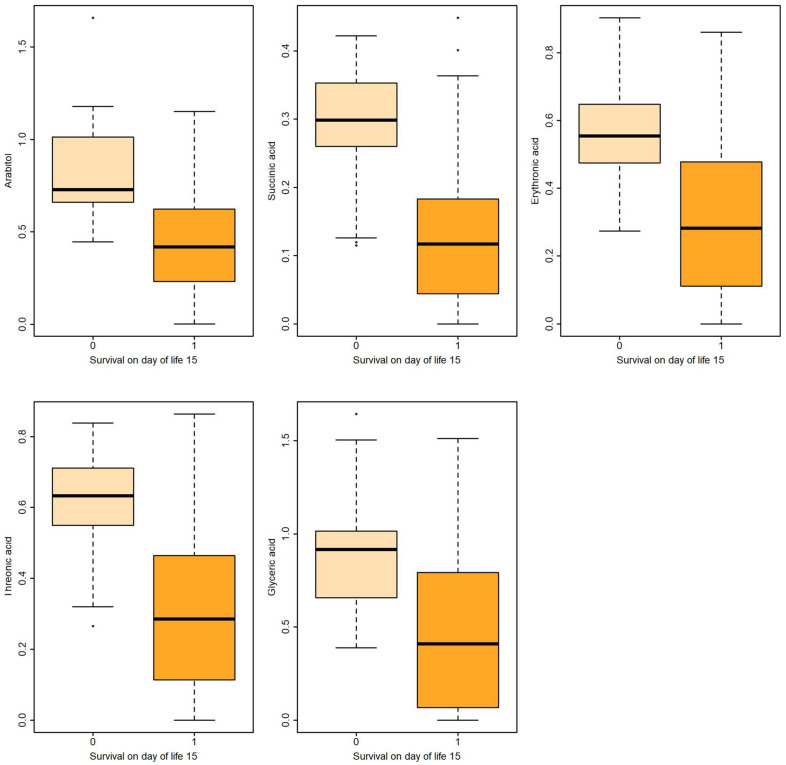
Boxplots of metabolites associated with survival on DOL 15 both in uni- and multivariable logistic regression.

**Figure 2:**
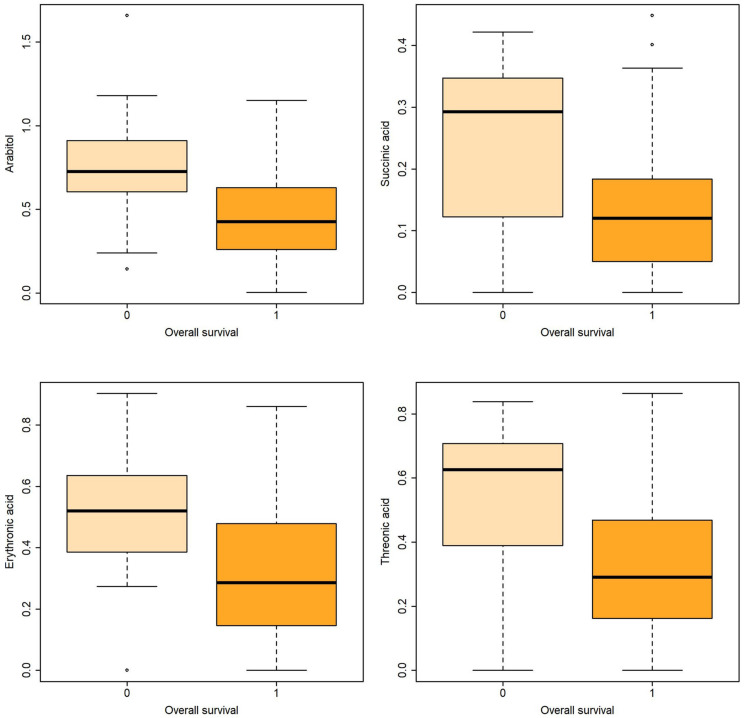
Boxplots of metabolites associated with overall survival both in uni- and multivariable logistic regression.

**Figure 3:**
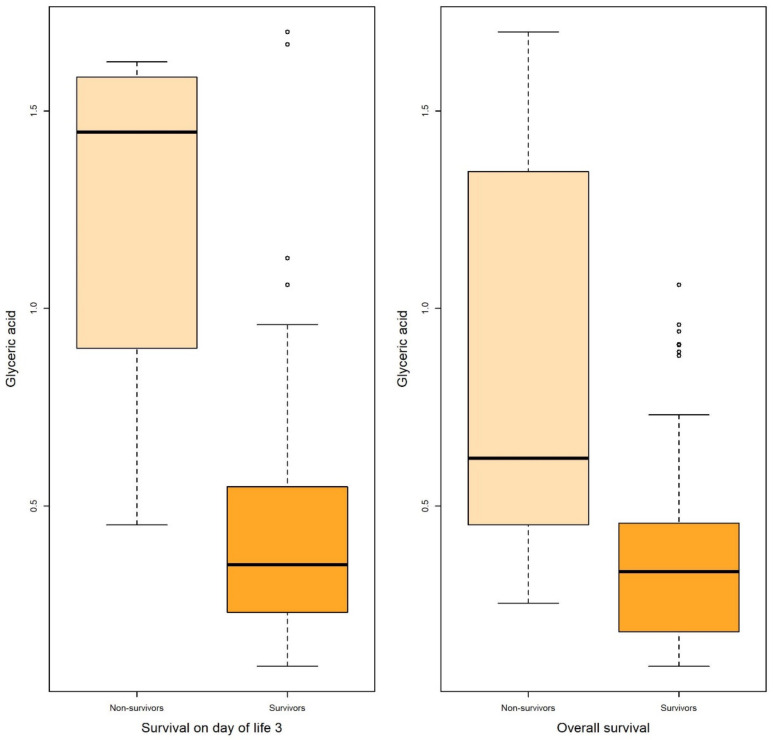
Boxplots of glyceric acid associated with survival on DOL 3 and overall survival in both uni- and multivariable logistic regression.

**Table 1:** Perinatal and neonatal characteristics in survivors and non-survivors in the first 3 and 15 days of life and up to hospital discharge.

	Survivors D3	Non-Survivors D3	P1	Survivors D15	Non-Survivors D15	P2	Overall Survivors	Overall Non-Survivors	P3
**Count**	**90**	**6**		**78**	**18**		**75**	**21**	
Gestational age (weeks)	28.5 ± 2.1	25.1 ± 2.3	<0.001	29 ± 1.6	25.2 ± 1.9	<0.001	29 ± 1.6	25.7 ± 2.3	<0.001
Birth weight (g)	1172 ± 342	770 ± 235	<0.001	1237 ± 309	756 ± 230	<0.001	1248 ± 305	783 ± 242	<0.001
SGA	7 (7.8)	1 (16.7)	0.415	6 (7.7)	2 (11.1)	0.642	5 (6.7)	3 (14.3)	0.367
Male sex	36 (40)	4 (66.7)	0.231	32 (41)	8 (44.4)	0.797	31 (41.3)	9 (42.9)	1
Multiple gestation	29 (32.2)	0 (0)	0.173	27 (34.6)	2 (11.1)	0.085	27 (36)	2 (9.5)	0.029
Hypertension/pregnancy-induced hypertension	11 (12.2)	1 (16.7)	0.511	11 (14.1)	1 (9.1)	0.511	10 (13.3)	2 (11.1)	1
Prenatal steroids	87 (96.7)	5 (83.3)	0.231	75 (96.2)	17 (94.4)	0.571	72 (96)	20 (95.2)	1
Maternal MgSO_4_ administration	55 (61.8)	3 (50)	1	45 (57.6)	13 (72.2)	0.270	43 (58.1)	15 (75)	0.202
Mode of delivery—CS	80 (89.9)	5 (83.3)	0.497	71 (92.2)	14 (77.8)	0.091	68 (91.9)	17 (81)	0.220
Chorioamnionitis (clinical or histological)	46 (51.1)	3 (50)	1	37 (47.4)	12 (66.7)	0.192	37 (49.3)	12 (57.1)	0.624
Intubation in the delivery room	30 (33.3)	6 (100)	0.002	22 (28.2)	14 (77.8)	<0.001	22 (29.3)	14 (66.7)	0.004
Apgar score at 1 min	6.3 ± 1.9	4.4 ± 2.4	0.038	6.4 ± 1.9	5.3 ± 2.2	0.063	6.4 ± 1.9	5.6 ± 2.1	0.197
Apgar score at 5 min	8.1 ± 0.9	7.2 ± 0.4	0.005	8.2 ± 0.9	7.4 ± 0.5	<0.001	8.2 ± 0.9	7.6 ± 0.7	0.003
SNAPPE-II score	22.1 ± 22.6	77.5 ± 22	0.003	17.9 ± 17.1	58.7 ± 30.2	<0.001	17.4 ± 17	54.3 ± 30.5	<0.001
RDS	60 (66.6)	6 (100)	0.173	51 (65.4)	15 (83.3)	0.083	48 (64)	18 (85.7)	0.029
Surfactant treatment	53 (58.9)	6 (100)	0.079	43 (55.1)	16 (88.9)	0.008	40 (46.7)	19 (90.5)	0.002
IMV in first 3 DOL IMV first 14 DOL IMV during NICU stay	42 (46.7) N/A N/A	6 (100) N/A N/A	0.026	N/A 19 (24.4) N/A	N/A 10 (55.6) N/A	0.020	N/A N/A 31 (41.3)	N/A N/A 21 (100)	<0.001
Air-leak syndromes	7 (7.8)	4 (66.7)	<0.001	3 (3.8)	8 (44.4)	<0.001	3 (4)	8 (38.1)	<0.001
IVH 3-4	16 (17.8)	2 (33.3)	0.313	7 (9)	11 (61.1)	<0.001	6 (89)	12 (51.7)	<0.001
Confirmed EOS	1 (1.1)	1 (16.7)	0.112	0 (0)	2 (11.1)	0.034	0 (0)	2 (9.5)	0.046
Confirmed LOS	N/A	N/A		27 (34.6)	5 (27.8)	0.782	25 (33.3)	7 (33.3)	1
Inotropes in first 3 DOL Inotropes in first 14 DOL Inotropes during NICU stay	22 (24.4) N/A N/A	6 (100) N/A N/A	<0.001	N/A 13 (16.7) N/A	N/A 16 (88.9) N/A	<0.001	N/A N/A 11 (14.7)	N/A N/A 18 (87.5)	<0.001
Drug treatment for PDA	25 (27.8)	0 (0)	0.334	18 (23.1)	7 (38.9)	0.232	15 (20)	10 (47.6)	0.022
NEC (all stages)	N/A	N/A		17 (21.8)	2 (11.1)	0.512	15 (20)	4 (19)	1
Surgical NEC	N/A	N/A		2 (2.6)	1 (5.6)	0.468	1 (1.3)	2 (9.5)	0.120
BPD	N/A	N/A		N/A	N/A		38 (50.7)	1 (4.8)	<0.001

Quantitative variables are shown as means ± SDs, and και quality variables are shown as numbers and % (percentage) within parenthesis. P1, P2 and P3: *p*-value is referred to the comparison between survivors and non-survivors at the respective time points. BPD: bronchopulmonary dysplasia; CS: cesarian section; DOL: day of life; EOS: early-onset sepsis; IMV: invasive mechanical ventilation; IVH: intraventricular hemorrhage; LOS: late-onset sepsis; N/A: non-applicable; NEC: necrotizing enterocolitis; NICU: neonatal intensive care unit; PDA: patent ductus arteriosus; RDS: respiratory distress syndrome; SGA: small for gestational age.

**Table 2:** AUC values of the studied perinatal and neonatal variables with respect to binary outcome endpoints.

Survival	DOL 3		DOL 15		Overall	
	AUC	*p*-Value	AUC	*p*-Value	AUC	*p*-Value
Gestational age	0.853	0.004	0.919	0.000	0.869	0.003
Birth weight	0.833	0.006	0.895	0.000	0.882	0.000
SGA	0.544	0.716	0.517	0.822	0.544	0.716
Sex	0.633	0.276	0.517	0.822	0.508	0.915
Multiple gestation	0.661	0.188	0.618	0.121	0.632	0.065
Hypertension/pregnancy induced hypertension	0.537	0.783	0.502	0.983	0.512	0.875
Prenatal steroids	0.567	0.586	0.509	0.910	0.504	0.958
Maternal MgSO_4_ administration	0.509	0.946	0.590	0.246	0.584	0.248
Mode of delivery-CS	0.567	0.586	0.509	0.910	0.504	0.958
Chorioamnionitis (clinical or histological)	0.506	0.122	0.596	0.205	0.539	0.586
Intubation in the delivery room	0.833	0.006	0.748	0.001	0.689	0.001
Apgar at 1 min	0.771	0.042	0.660	0.004	0.597	0.186
Apgar at 5 min	0.837	0.012	0.811	0.000	0.754	0.001
SNAPPE-II score	0.951	0.000	0.889	0.000	0.867	0.000
RDS	0.888	0.002	0.704	0.009	0.744	0.001
Surfactant treatment	0.746	0.044	0.701	0.008	0.736	0.001
IMV in first 3 DOL IMV in first 14 DOL IMV during NICU stay	0.767 N/A N/A	0.029	N/A 0.656 N/A	0.04	N/A N/A 0.759	0.000
Air-leak syndromes	0.794	0.016	0.703	0.007	0.670	0.017
IVH 3-4	0.578	0.525	0.761	0.010	0.746	0.001
Confirmed EOS	0.490	0.934	N/A		N/A	
Confirmed LOS	N/A	N/A	0.562	0.417	0.510	0.887
Inotropes in first 3 DOL Inotropes in first 14 DOL Inotrope during NICU stay	0.878 N/A N/A	0.002	N/A 0.861 N/A	0.000	N/A N/A 0.855	0.000
Drug treatment for PDA	0.639	0.256	0.579	0.297	0.638	0.054
NEC (all stages)	N/A		0.543	0.567	0.509	0.905
Surgical NEC	N/A		0.485	0.844	0.459	0.568
BPD	N/A		N/A		0.733	0.001

AUC: area under the curve; BPD: bronchopulmonary dysplasia; CS: cesarian section; DOL: day of life; EOS: early-onset sepsis; IMV: invasive mechanical ventilation; IVH: intraventricular hemorrhage; LOS: late-onset sepsis; N/A: non-applicable; NEC: necrotizing enterocolitis; NICU: neonatal intensive care unit; PDA: patent ductus arteriosus; RDS: respiratory distress syndrome; SGA: small for gestational age.

**Table 3:** Descriptive and AUC values of gastric fluid metabolites with significant prognostic value at specific time points throughout the study.

Metabolite	Outcome	N	Mean	SD	SE	Q0.25	Q0.5	Q0.75	AUC	AUC 95% CI
**Survival on Day of Life 15**										
Arabitol (HMDB0000568)	Non-survival	13	0.842	0.323	0.089	0.661	0.729	1.012	0.863	[0.769,0.956]
	Survival	60	0.425	0.261	0.034	0.236	0.419	0.6216		
Succinic acid (HMDB0000254)	Non-survival	13	0.285	0.105	0.029	0.259	0.298	0.352	0.824	[0.708,0.940]
	Survival	60	0.129	0.112	0.014	0.044	0.117	0.182		
Erythronic acid (HMDB0000613)	Non-survival	13	0.580	0.192	0.053	0.474	0.554	0.647	0.841	[0.734,0.948]
	Survivors	60	0.288	0.215	0.027	0.124	0.282	0.477		
Threonic (HMDB0000943)	Non-survival	13	0.605	0.173	0.048	0.551	0.633	0.711	0.845	[0.737,0.953]
	Survival	60	0.304	0.234	0.031	0.129	0.285	0.461		
Glyceric acid (HMDB0000139)	Non-survival	13	0.910	0.369	0.102	0.656	0.916	1.014	0.786	[0.668,0.904]
	Survival	60	0.483	0.416	0.053	0.081	0.408	0.780		
**Overall survival**										
Arabitol	Non-survival	15	0.755	0.376	0.097	0.604	0.725	0.911	0.771	[0.626,0.916]
	Survival	58	0.433	0.262	0.034	0.265	0.426	0.626		
Succinic acid	Non-survival	15	0.247	0.139	0.036	0.122	0.292	0.347	0.737	[0.575,0.900]
	Survival	58	0.133	0.112	0.014	0.051	0.120	0.183		
Erythronic acid	Non-survival	15	0.502	0.271	0.069	0.385	0.519	0.635	0.737	[0.575,0.900]
	Survival	58	0.297	0.212	0.027	0.147	0.285	0.478		
Threonic acid	Non-survival	15	0.524	0.266	0.068	0.388	0.626	0.707	0.741	[0.577,0.905]
	Survival	58	0.315	0.231	0.031	0.164	0.291	0.466		

Corresponding results before and after adjusting for the characteristics in Table 2 are shown in Table 4 and are illustrated with boxplots in Figure 1 and Figure 2. Multiple logistic regression was used to adjust for perinatal/neonatal characteristics associated with the outcome, such as gestational age, birthweight, SNAPPE-II score, respiratory distress syndrome, air-leak syndromes and intraventricular hemorrhage grades 3–4.

**Table 4:** Significant gastric fluid metabolites associated with survival on DOL 15 and overall survival.

	Univariate Analysis			Multivariable Analysis		
Variable	OR	95% CI	*p*-Value	OR	95% CI	*p*-Value
	**Survival on Day of Life 15**					
Arabitol	0.175	[0.051,0.425]	<0.01	0.043	[0.0007,0.311]	0.032
Succinic acid	0.259	[0.112,0.517]	<0.01	0.004	[0.0013,0.242]	0.011
Erythronic acid	0.194	[0.060,0.453]	<0.01	0.076	[0,0.012]	0.011
Threonic acid	0.212	[0.076,0.474]	<0.01	0.0000002	[0,0.003]	0.024
Glyceric Acid	0.476	[0.244,0.839]	0.017	0.197	[0.043,0.787]	0.020
	**Overall survival**					
Arabitol	0.004	[0.0001,0.066]	<0.01	0.005	[0.00005,0.123]	<0.01
Succinic Acid	0.004	[0,0.005]	<0.01	0.000025	[0.000000003,0.026]	<0.01
Threonic acid	0.001	[0,0.036]	<0.01	0.005324	[0.00006,0.202]	<0.01
Erythronic acid	0.0021	[0,0.051]	<0.01	0.005208	[0.00005,0.197]	<0.01

**Table 5:** Descriptive and AUC values of the urine metabolites significantly associated with endpoints of interest.

Metabolite	Outcome	N	Mean	SD	SE	Q0.25	Q0.5	Q0.75	AUC	AUC 95% CI
	Survival	68	0.611	0.971	0.117	0.129	0.274	0.569		
	**Survival on Day of Life 3**									
Glyceric acid	Non-survival	4	1.242	0.540	0.270	1.122	1.446	1.566	0.896	[0.736,1.000]
	Survival	82	0.437	0.318	0.035	0.231	0.351	0.543		
	**Survival on Day of Life 15**									
Glyceric acid	Non-survival	15	0.893	0.547	0.141	0.491	0.601	1.446	0.809	[0.689,0.929]
	Survival	71	0.3867	0.244	0.028	0.190	0.341	0.466		
Proline	Non-survival	15	2.287	2.781	0.718	0.139	1.126	3.4261	0.681	[0.494–0.869]
	Survival	71	0.601	0.953	0.113	0.130	0.272	0.591		
	**Overall Survival**									
Glyceric acid	Non-survival	18	0.837	0.517	0.122	0.471	0.620	1.291	0.805	[0.696–0.914]
	Survival	68	0.379	0.244	0.029	0.182	0.333	0.454		
Proline HMDB0000162	Non-survival	18	1.965	2.632	0.621	0.166	0.841	3.008	0.648	[0.481–0.815]
	Survival	68	0.611	0.971	0.117	0.129	0.274	0.569		

However, only glyceric acid was significantly associated with 3 DOL and overall survival after adjusting for baseline characteristics (illustrated with boxplots in Figure 3), while results were marginal for 15 DOL survival (Table 6).

**Table 6:** Significant urine glyceric acid associated with survival on DOL 15 and overall survival.

	Univariate Analysis			Multivariable Analysis		
Variable	OR	95% CI	*p*-Value	OR	95% CI	*p*-Value
	**Survival on Day of Life 3**					
Glyceric acid	0.287	[0.136,0.518]	<0.01	0.267	[0.110,0.521]	<0.01
	**Survival on Day of Life 15**					
Glyceric acid	0.287	[0.136,0.518]	<0.01	0.078	[0.004,0.893]	0.052
	**Overall Survival**					
Glyceric acid	0.297	[0.144, 0.530]	<0.01	0.056	[0.004,0.403]	<0.01

## Data Availability

Not applicable.

## Supplementary Materials

The following supporting information can be downloaded at: https://www.mdpi.com/article/10.3390/metabo13060708/s1, Figure S1: Separation of survivors vs. non-survivors (DOL 15) based on metabolic profiles of gastric fluid using PLS-DA; Figure S2: Hierarchical cluster analysis of survivors vs. non-survivors (DOL 15) based on metabolic profiles of gastric fluid. Patterns of visual separability are apparent; Figure S3: Separation of survivors vs. non-survivors (overall survival) based on metabolic profiles of gastric fluid using PLS-DA; Figure S4: Hierarchical cluster analysis of survivors vs. non-survivors (overall survival) based on metabolic profiles of gastric fluid. Patterns of visual separability are apparent; Figure S5: Separation of survivors vs. non-survivors (DOL 3) based on metabolic profiles of urine using PLS-DA; Figure S6: Hierarchical cluster analysis results shown as a heatmap of survivors vs. non-survivors (DOL 3) based on metabolic profiles of gastric fluid. Patterns of visual separability exist; Figure S7: Separation of survivors vs. non-survivors (DOL15) based on metabolic profiles of urine using PLS-DA; Figure S8: Hierarchical cluster analysis results shown as a heatmap of survivors vs. non-survivors (DOL15) based on metabolic profiles of gastric fluid. Patterns of visual separability exist; Figure S9: Separation of survivors vs. non-survivors (overall survival) based on metabolic profiles of urine using PLS-DA; Figure S10: Hierarchical cluster analysis results shown as a heatmap of survivors vs. non-survivors (overall survival) based on metabolic profiles of gastric fluid. Patterns of visual separability exist. Refs. [43,44,45,46,47,48,49,50] were listed in Supplementary Materials.
