# Supplementary material for: A Cohort Study of Gastric Fluid and Urine Metabolomics for the Prediction of Survival in Severe Prematurity
Source: Metabolites. 2023 May 30;13(6):708. doi: 10.3390/metabo13060708 (PMC10304811; doi:10.3390/metabo13060708)
Supplement: Supplementary file 1 [file metabolites-13-00708-s001.zip › metabolites-2400388-supplementary.pdf]

Definitions

Bronchopulmonary dysplasia was considered in infants who were receiving supplemental oxygen at 36 weeks of postmenstrual age [1].

Gestational age was determined with the use of the best obstetrical estimate based on the date of the last menstrual period and/or prenatal ultrasonography. If the best obstetrical estimate was unavailable or uncertain, gestational age was determined on the basis of the neonatologist's estimate with the use of physical examination criteria, such as the New Ballard Score [2].

Necrotizing enterocolitis was defined as Bell's stages II to III, according to the modified Bell's classification (with scores ranging from I to III and higher scores indicating greater severity of disease) [3]. **Surgical NEC** included cases requiring surgical management of the disease.

Respiratory distress syndrome was considered in preterm infants who developed respiratory insufficiency soon after birth and had lung X-ray findings compatible with pulmonary immaturity and surfactant deficiency [4].

Sepsis was classified according to the time of its appearance after birth as **early-onset** (≤72 hours of life) or **late-onset** (>72 hours of life) [5,6]. Cases of **confirmed** early- or late-onset **sepsis** had positive blood cultures for microbes or fungi, and cases of **possible sepsis** had clinical and laboratory evidence of sepsis but negative blood cultures.

Intraventricular hemorrhage was defined according to the criteria by Papile et al. [7].

Small for gestational age was defined as birth weight below the 10th percentile on the Fenton preterm growth charts [8].

References

1. Jobe, A.H.; Bancalari, E. Bronchopulmonary Dysplasia. *Am. J. Respir. Crit. Care Med.* **2001**, *163*, 1723–1729, doi:10.1164/ajrccm.163.7.2011060.
2. Ballard, J.L.; Khoury, J.C.; Wedig, K.; Wang, L.; Eilers-Walsman, B.L.; Lipp, R. New Ballard Score, Expanded to Include Extremely Premature Infants. *J. Pediatr.* **1991**, *119*, 417–423, doi:10.1016/s0022-3476(05)82056-6.
3. Walsh, M.C.; Kliegman, R.M. Necrotizing Enterocolitis: Treatment Based on Staging Criteria. *Pediatr. Clin. North Am.* **1986**, *33*, 179–201, doi:10.1016/s0031-3955(16)34975-6.
4. Sweet, D.G.; Carnielli, V.P.; Greisen, G.; Hallman, M.; Klebermass-Schrehof, K.; Ozek, E.; Te Pas, A.; Plavka, R.; Roehr, C.C.; Saugstad, O.D.; et al. European Consensus Guidelines on the Management of Respiratory Distress Syndrome: 2022 Update. *Neonatology* **2023**, *120*, 3–23, doi:10.1159/000528914.
5. Dong, Y.; Speer, C.P. Late-Onset Neonatal Sepsis: Recent Developments. *Arch. Dis. Child. Fetal Neonatal Ed.* **2015**, *100*, F257-263, doi:10.1136/archdischild-2014-306213.
6. Cortese, F.; Scicchitano, P.; Gesualdo, M.; Filaninno, A.; De Giorgi, E.; Schettini, F.; Laforgia, N.; Ciccone, M.M. Early and Late Infections in Newborns: Where Do We Stand? A Review. *Pediatr. Neonatol.* **2016**, *57*, 265–273, doi:10.1016/j.pedneo.2015.09.007.
7. Papile, L.A.; Burstein, J.; Burstein, R.; Koffler, H. Incidence and Evolution of Subependymal and Intraventricular Hemorrhage: A Study of Infants with Birth Weights Less than 1,500 Gm. *J. Pediatr.* **1978**, *92*, 529–534, doi:10.1016/s0022-3476(78)80282-0.
8. Fenton, T.R.; Kim, J.H. A Systematic Review and Meta-Analysis to Revise the Fenton Growth Chart for Preterm Infants. *BMC Pediatr.* **2013**, *13*, 59, doi:10.1186/1471-2431-13-59.

Figures S1–S10

Gastric Fluid

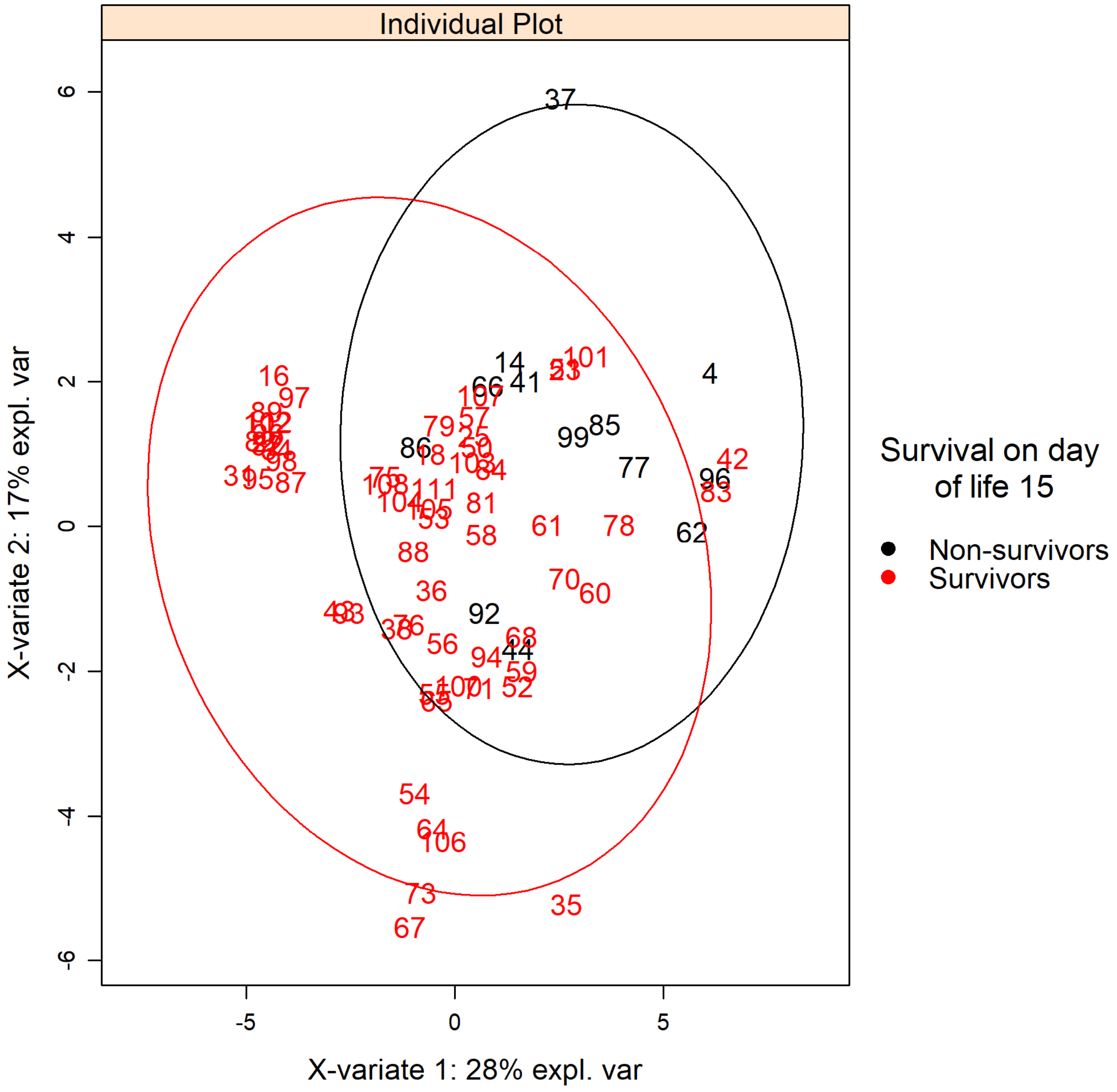

Figure S1. Separation of survivors vs. non-survivors (DOL 15) based on metabolic profiles of gastric fluid using PLS-DA.

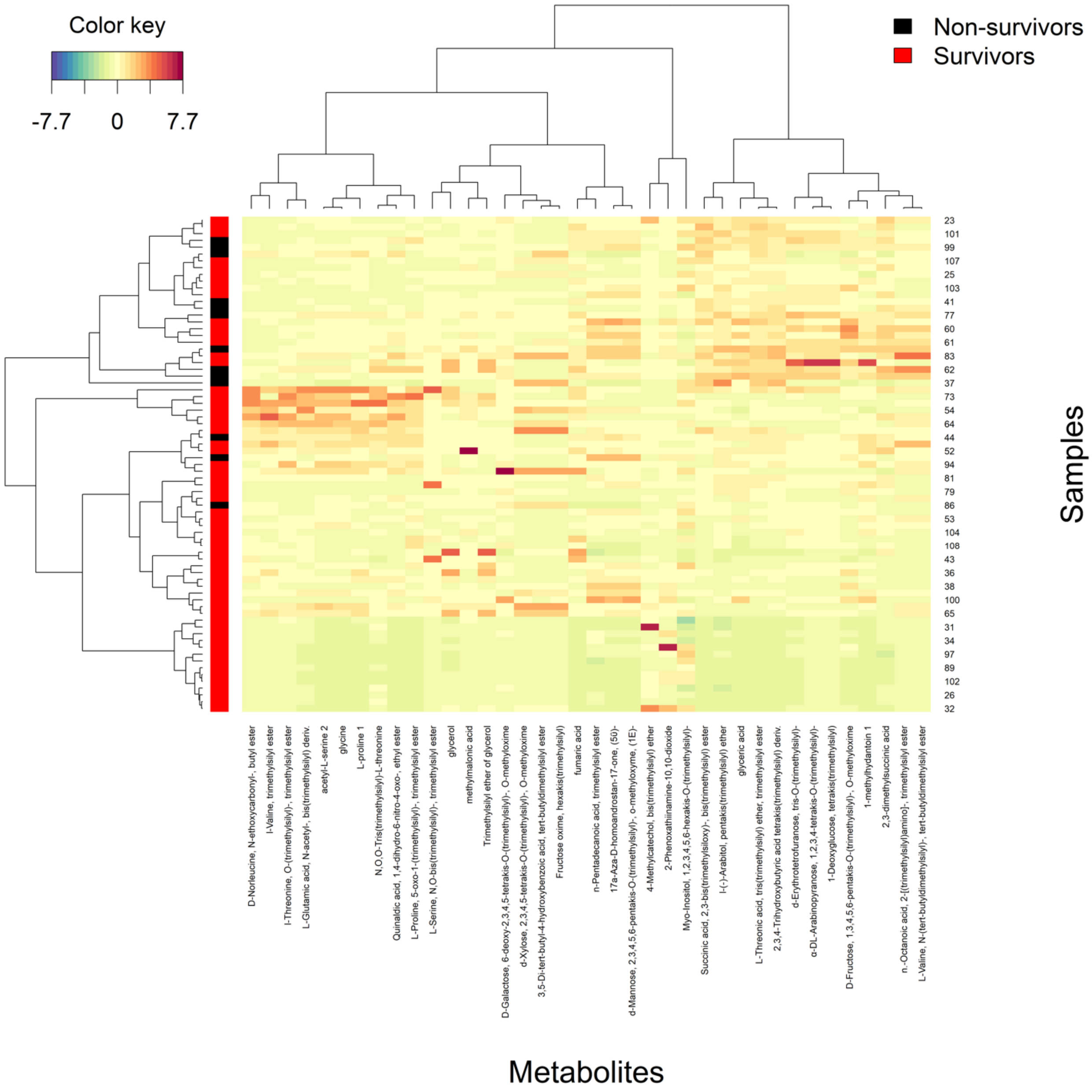

Figure S2. Hierarchical cluster analysis of survivors vs. non-survivors (DOL 15) based on metabolic profiles of gastric fluid. Patterns of visual separability are apparent.

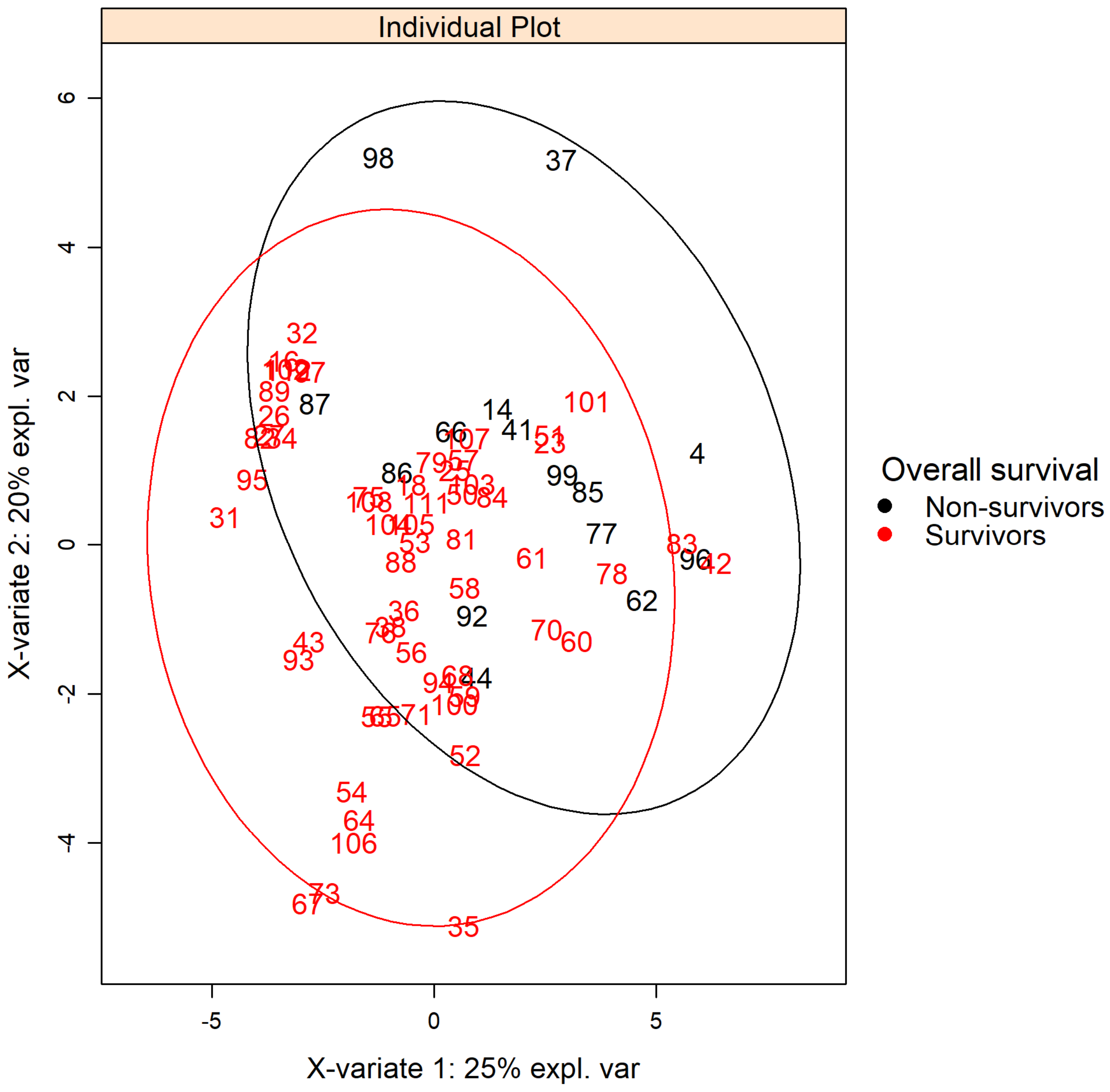

Figure S3. Separation of survivors vs. non-survivors (overall survival) based on metabolic profiles of gastric fluid using PLS-DA.

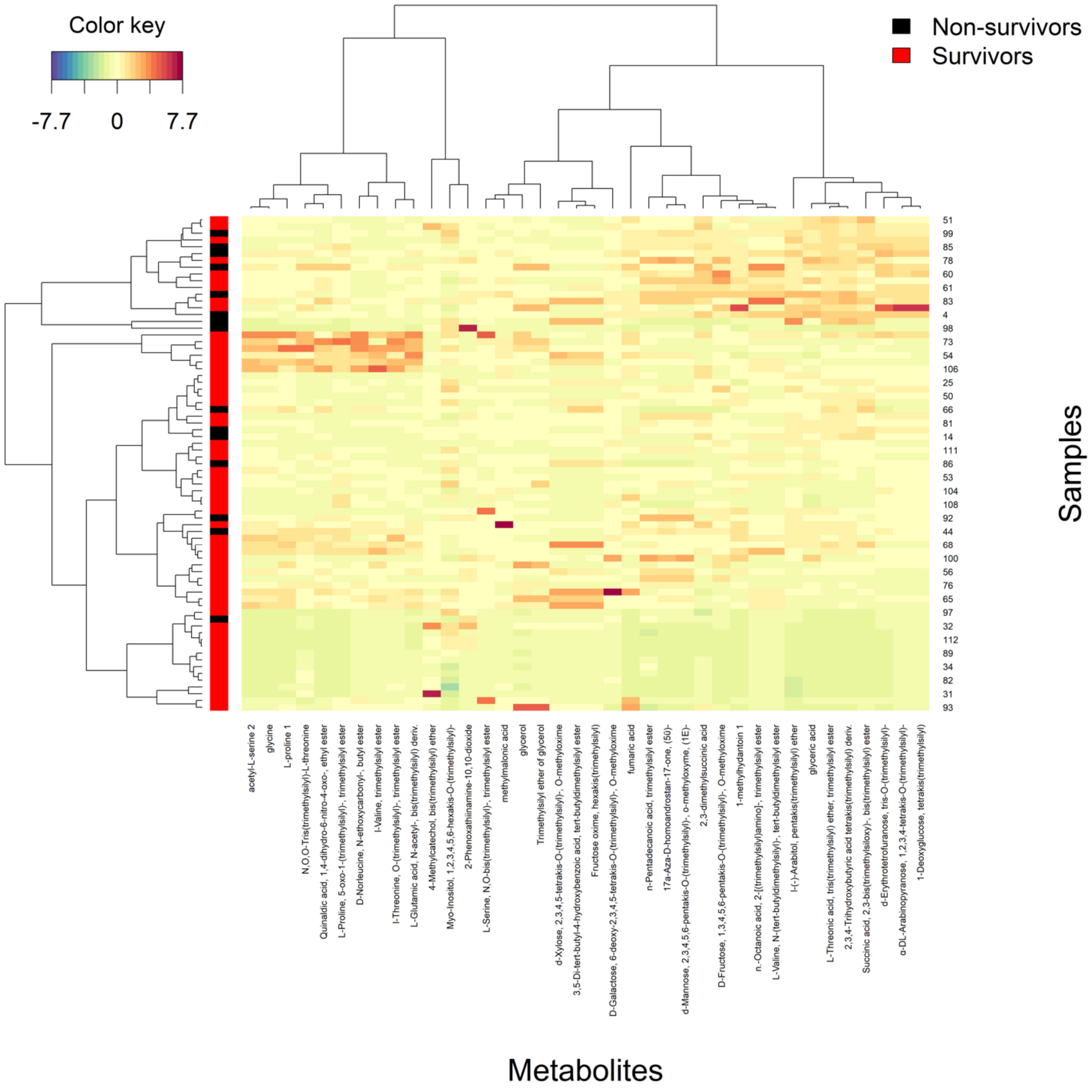

Figure S4. Hierarchical cluster analysis of survivors vs. non-survivors (overall survival) based on metabolic profiles of gastric fluid. Patterns of visual separability are apparent.

Urine

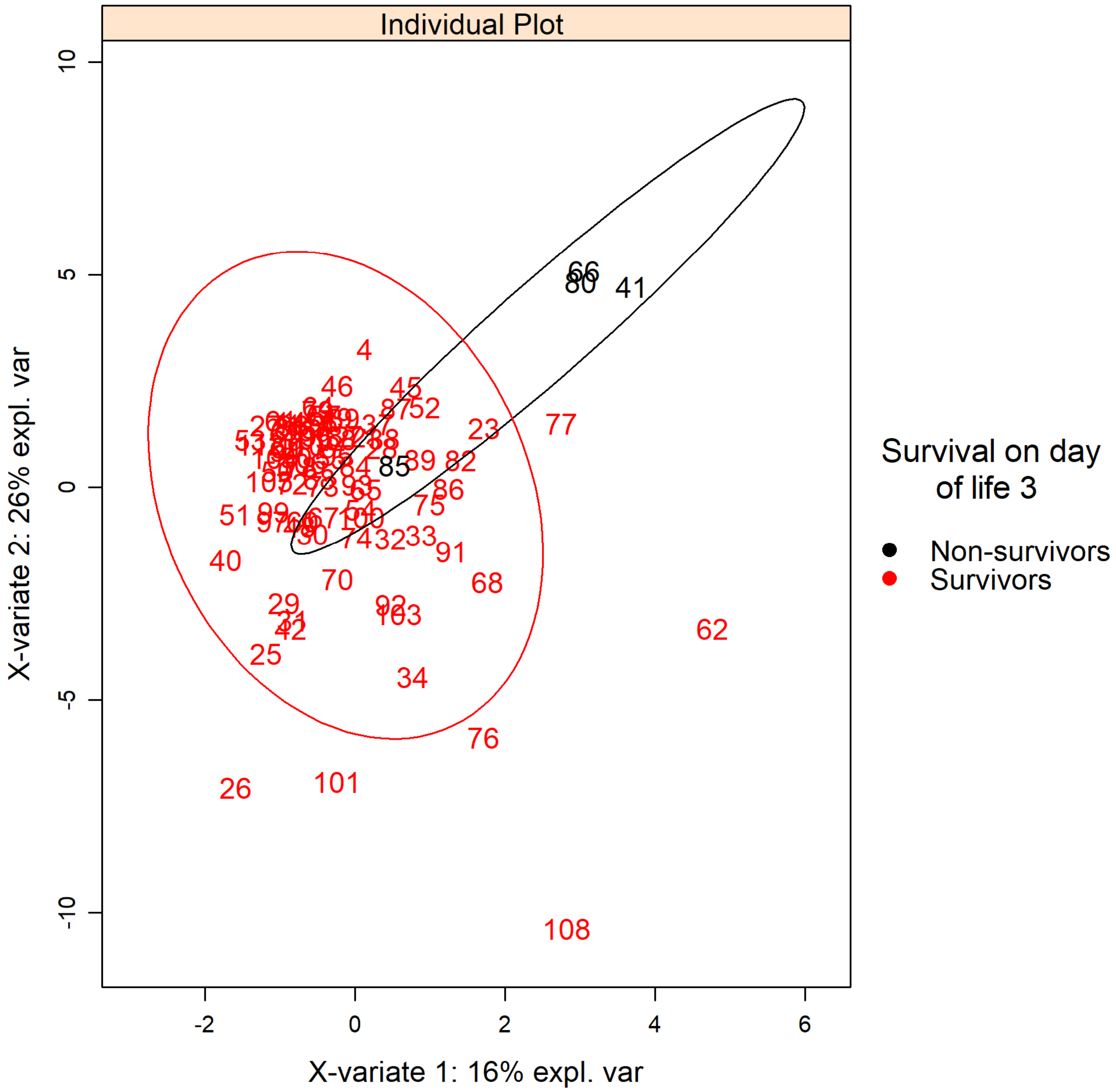

Figure S5. Separation of survivors vs. non-survivors (DOL 3) based on metabolic profiles of urine using PLS-DA.

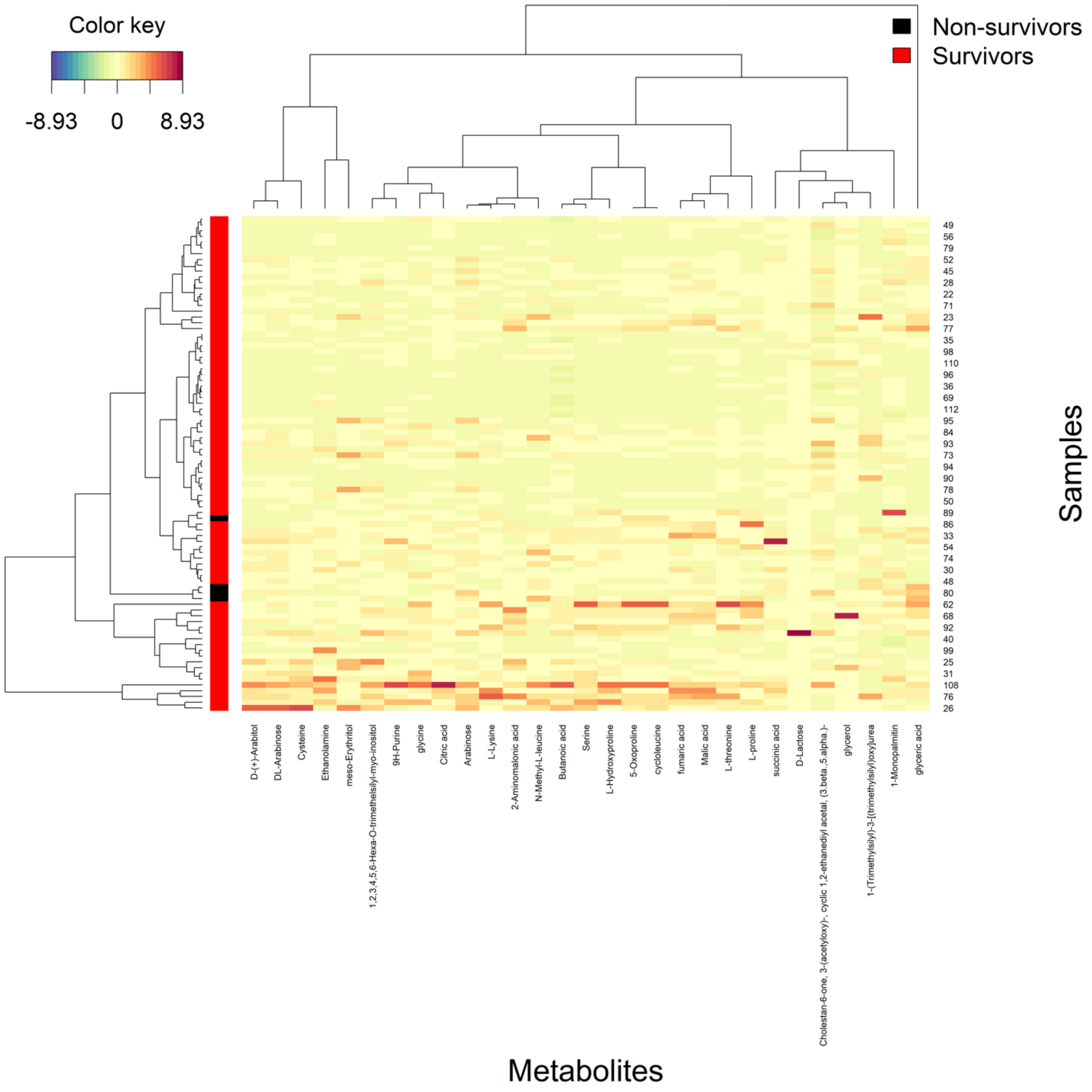

Figure S6. Hierarchical cluster analysis results shown as a heatmap of survivors vs. non-survivors (DOL 3) based on metabolic profiles of gastric fluid. Patterns of visual separability exist.

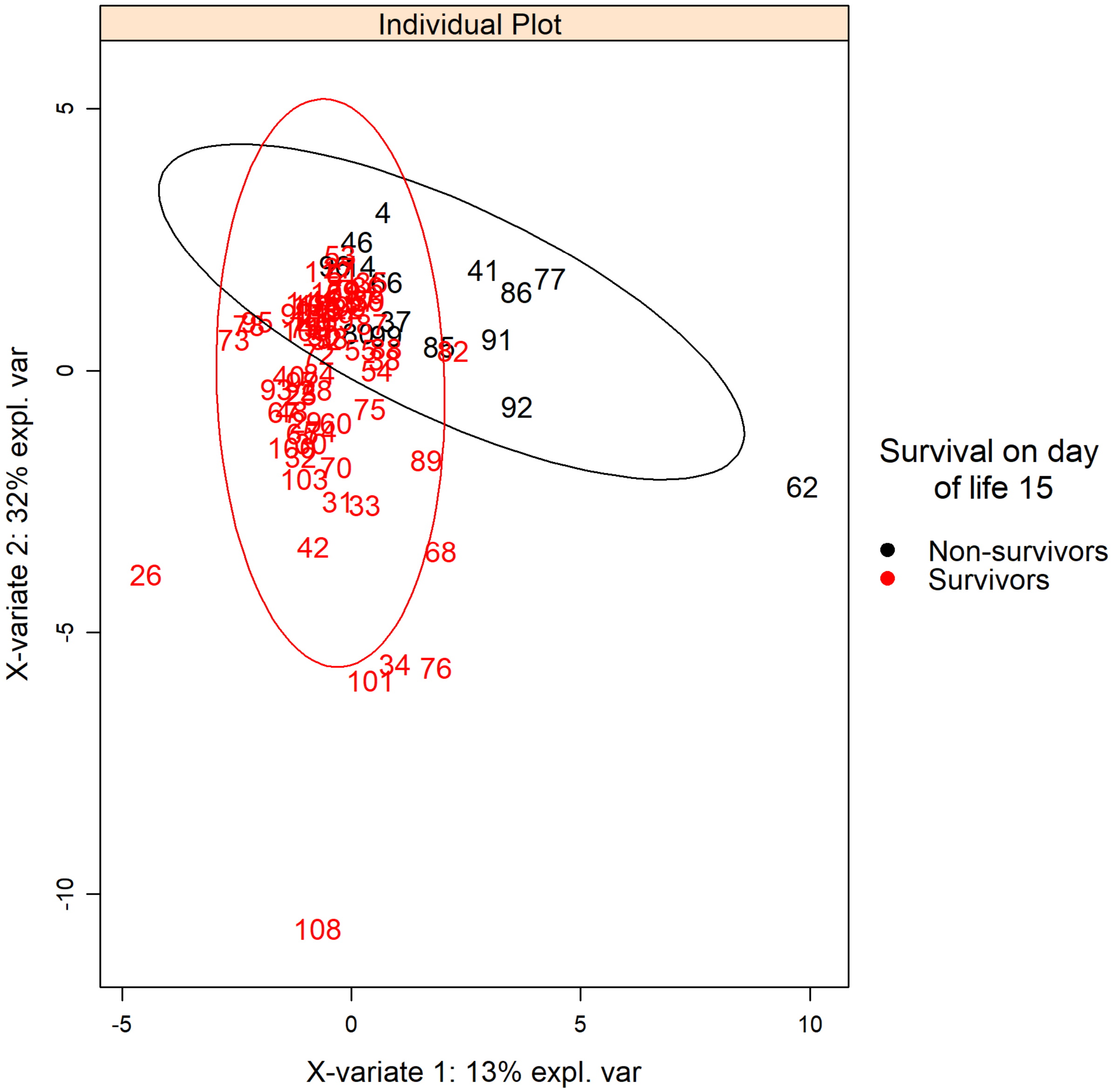

Figure S7. Separation of survivors vs. non-survivors (DOL15) based on metabolic profiles of urine using PLS-DA.

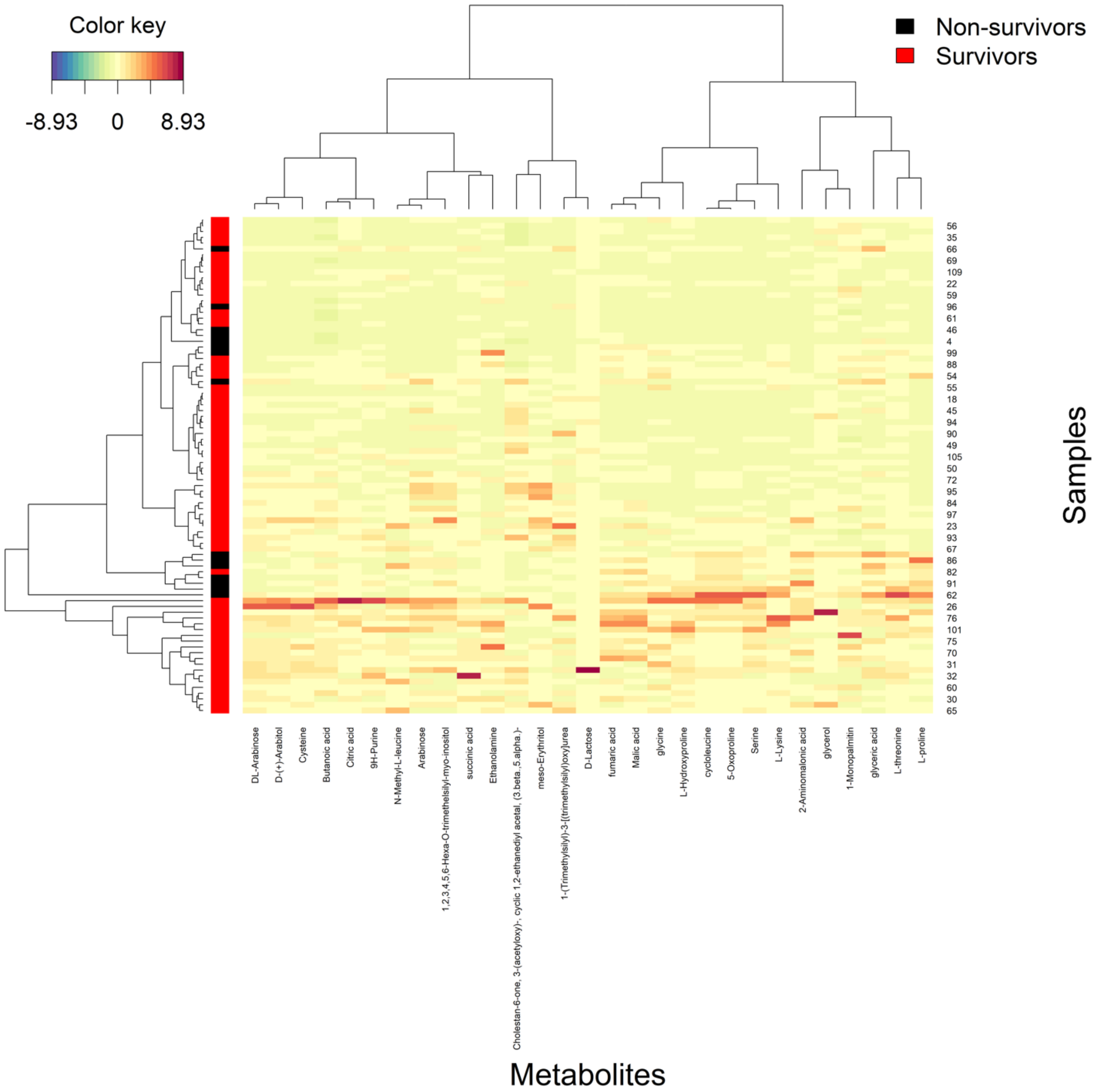

Figure S8. Hierarchical cluster analysis results shown as a heatmap of survivors vs. non-survivors (DOL15) based on metabolic profiles of gastric fluid. Patterns of visual separability exist.

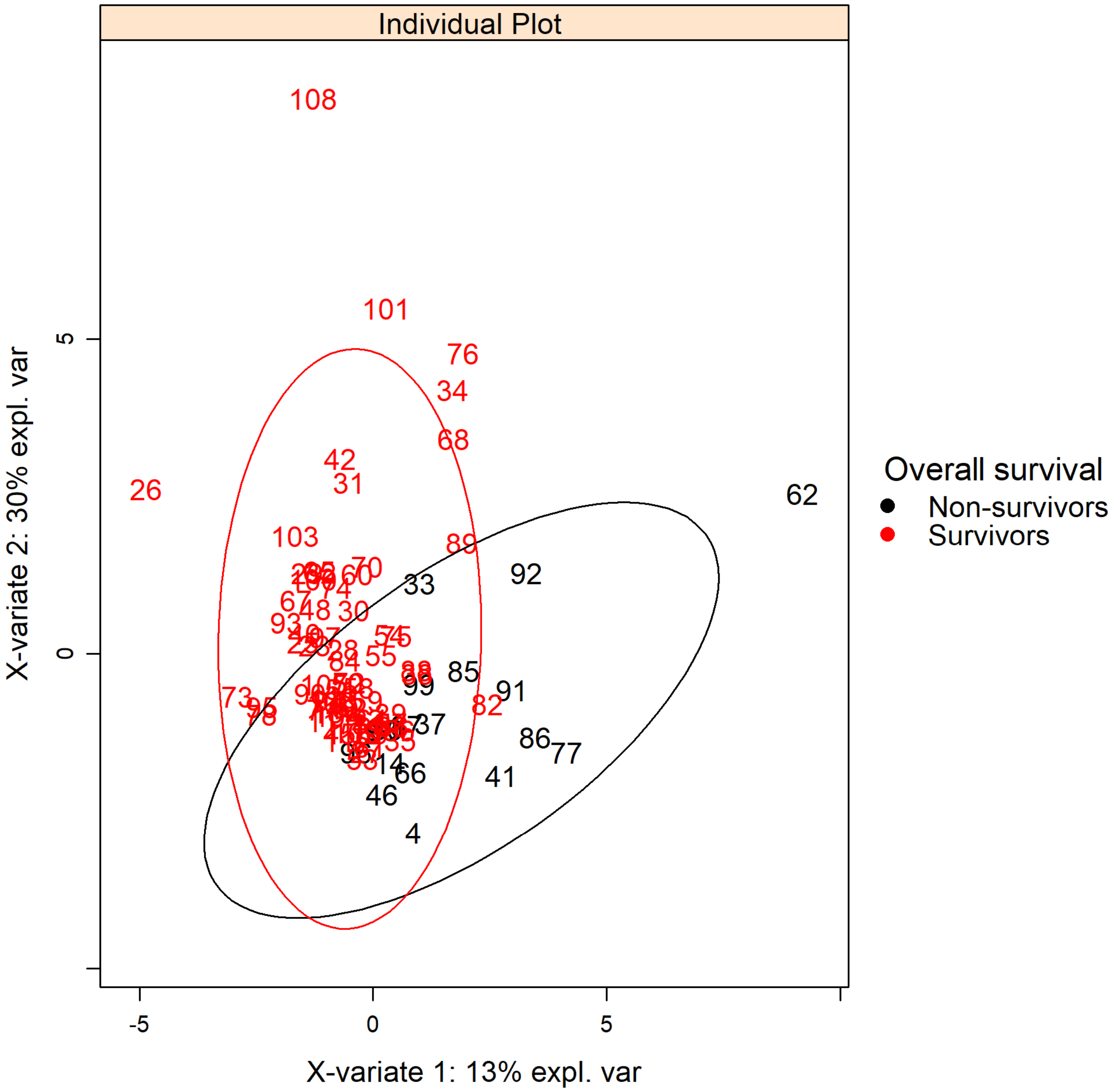

Figure S9. Separation of survivors vs. non-survivors (overall survival) based on metabolic profiles of urine using PLS-DA.

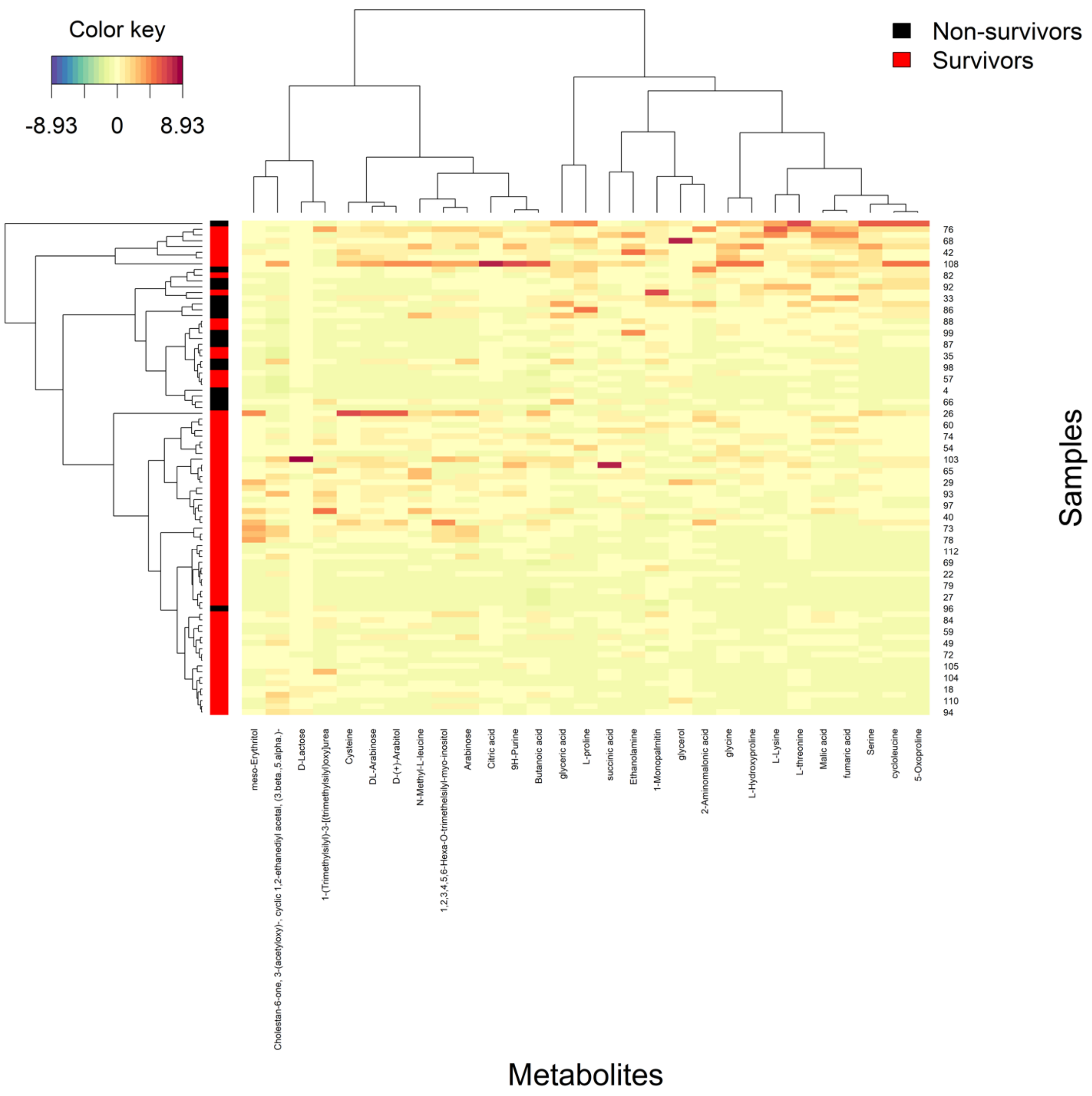

Figure S10. Hierarchical cluster analysis results shown as a heatmap of survivors vs. non-survivors (overall survival) based on metabolic profiles of gastric fluid. Patterns of visual separability exist.
